# Knowledge, attitude, and practice regarding antibiotic use and resistance among university students in Ethiopia: a cross-sectional survey study

**DOI:** 10.1080/20523211.2025.2587455

**Published:** 2025-11-19

**Authors:** Samuel Berihun Dagnew, Tilaye Arega Moges, Getu Tesfaw Addis, Getachew Yitayew Tarekegn, Abate Wondesen Tsige, Teklie Mengie Ayele, Fisseha Nigussie Dagnew, Samuel Agegnew Wondm

**Affiliations:** a Department of Clinical Pharmacy, College of Health Sciences, Debre Tabor University, Debre Tabor, Ethiopia; d Department of Pharmacology and Toxicology, College of Health Sciences, Debre Tabor University, Debre Tabor, Ethiopia; b Department of Social and Administrative Pharmacy, College of Health Sciences, Debre Tabor University, Debre Tabor, Ethiopia; e Department of Pharmacy, College of Health Sciences, Debre Markos University, Debre Markos, Ethiopia; c Department of Clinical Pharmacy, College of Health Sciences, Debre Berhan University, Debre Berhan, Ethiopia

**Keywords:** Knowledge, practice, attitude, antibiotic use, antibiotics resistance, Debre Tabor university students

## Abstract

**Background:**

Antibiotics are lifesaving drugs when used appropriately. On the other hand, inappropriate use causes antibiotic-resistant pathogenic bacteria to grow quickly, which has negative health effects such treatment failure, longer hospital stays, greater medical expenses, decreased efficacy, morbidity, and death. The aim of the study is to assess knowledge, attitude, and practice of antibiotic use and resistance among university students.

**Methods:**

A university-based cross-sectional survey study was conducted in Northwest Ethiopia from April 1 to June 30, 2022 among regular undergraduate students at Debre Tabor University. A stratified random sampling technique was used. The Statistical Package for Social Science for Windows version 27 was used to enter and analyze the data. The variables influencing knowledge, attitudes, and practices regarding the usage of antibiotics were evaluated using logistic regression. We used 95% confidence intervals and considered *p*-values less than 0.05 as significant.

**Results:**

In the end, 316 participants were included in the research. 176 (55.7%) of the participants were male, and 226 (71.5%) were in the 20–25 age range. About half 52.4% of the students showed good knowledge, 48.9% had a positive attitude, and 45.7% used antibiotics appropriately. More than half of the students (54.7%) demonstrated an adequate understanding of antibiotic resistance, while nearly half (45.2%) exhibited a positive attitude toward it. Factors influencing knowledge, attitudes, and practices (KAP) of antibiotic use were identified. Students from rural areas were 48% less likely to be knowledgeable than urban students (AOR = 0.526, 95% CI: 0.159–0.837, *p* = 0.012). Compared to health related majors, non-health majors had significantly lower knowledge (AOR = 0.660, 95% CI: 0.159–0.987, *p* = 0.012) and poorer practices (AOR = 0.551, 95% CI: 0.292–0.845, *p* = 0.013).

**Conclusion:**

The findings indicated insufficient knowledge, unfavourable attitudes, and inappropriate practices concerning antibiotic use and resistance. Targeted interventions may be implemented through structured meetings, educational materials, and comprehensive training programs.

## Background

Antibiotics are the most commonly prescribed drugs in developed and developing countries, despite the widespread prescription of treatments for non-communicable diseases (Organization, 2012; Sakr et al., 2020). These medications aid in treating bacterial infections by killing and inhibiting the growth bacteria in the body (Costelloe et al., 2010). Globally, antibiotics are essential for reducing the prevalence of infectious diseases (Costelloe et al., 2010; Ndihokubwayo et al., 2013). Antibiotics are still one of the most effective treatments for bacterial illnesses; therefore taking them alone is not the problem. Rather, the overuse or misuse of antibiotics can hasten the evolution of resistance (Battah, Halboup, et al., 2021; Sunusi et al., 2019). Antibiotics are drugs that either kill or stop the growth of bacteria in order to prevent or treat bacterial infections; they do not work against viral infections, and their abuse can lead to antibiotic resistance (Lessa & Sievert, 2023).

Antibiotic resistance (ABR) is a major global public health concern, closely associated with the unnecessary and inappropriate use of antibiotics (Costelloe et al., 2010). While ABR can occur naturally when bacteria are exposed to antibiotics (Organization, 2018), it is significantly accelerated when patients receive the correct treatment and dosage but fail to respond clinically or microbiologically (Cantón & Morosini, 2011). The World Health Organization (WHO) identifies ABR as a top global health threat, warning it could cause up to 10 million deaths annually by 2050 (Tornimbene et al., 2022). In 2019, ABR was linked to 4.95 million deaths, including 1.27 million directly. Sub-Saharan Africa had the highest mortality rate, with 24 deaths per 100,000 people (Tang et al., 2023).

Key contributors to ABR include overuse of antibiotics, lack of awareness regarding their indications and specificity, non-adherence to prescribed regimens, and self-medication (Laxminarayan et al., 2013; Ventola, 2015). The consequences are severe limiting treatment options, prolonging illness, increasing hospital stays, and escalating healthcare costs (Chai et al., 2022). Inappropriate antibiotic use, responsible for about 50% of global usage, remains the most common cause of resistance, allowing bacteria to adapt and rendering antibiotics ineffective (Organization, 2016). Ultimately, ABR results in treatment failure, increased morbidity and mortality, and undermines healthcare progress (Ndihokubwayo et al., 2013).

The high burden of infectious diseases, irrational antibiotic use, inadequate infection control policies, inadequate medication, inadequate knowledge of antimicrobial resistance, incorrect diagnosis, and a lack of laboratories for antibiotic susceptibility testing are the primary causes of the antimicrobial resistance crisis in developing nations (Organization, 2016; Seid & Hussen, 2018a). Antibiotic overuse or inappropriate use can lead to bacterial resistance, unfavourable side effects, and financial strain on national health systems (Abbo et al., 2013).

Preventing antibiotic resistance requires addressing key contributing factors such as inappropriate use, lack of knowledge, negative attitudes, unregulated medicine sales, and self-medication (Kong et al., 2021). Students should be heavily involved in minimising the overuse and abuse of antibiotics (Battah, Halboup, et al., 2021). However, studies reveal a high prevalence of improper antibiotic use among students, often linked to self-medication, limited knowledge of indications, pathogen specificity, and poor adherence to dosage regimens (Al Barakh et al., 2016; Sakr et al., 2020).

Globally and in Ethiopia, the misuse and overuse of antibiotics particularly through self-medication are major drivers of antibiotic resistance (ABR), especially in low – and middle-income countries (Bogale et al., 2019; Organization, 2015; Salam et al., 2023). In Ethiopia, self-medication and inappropriate use of antibiotics remain widespread, further contributing to the rise of resistance (Global burden of bacterial antimicrobial resistance in 2022: a systematic analysis, 2022). However, only a few studies have systematically examined the knowledge, attitudes, and practices (KAP) of Ethiopian university students regarding antibiotic use and resistance. Generating such locally relevant evidence is crucial to identify knowledge gaps, guide awareness programs, and strengthen both national and international efforts to combat ABR. This study, therefore, aims to assess the KAP of regular university students toward antibiotic use and resistance.

## Methods

### Study setting, period, and design

The study was conducted at Debre Tabor University (DTU), a public higher education institution in Ethiopia, between April 1 and June 30, 2022. The university comprises five faculties: the College of Health Sciences, College of Natural and Computational Sciences, College of Social Sciences and Humanities, College of Agriculture and Environmental Sciences, and College of Business and Economics. Study participants were selected from the College of Health Sciences, which includes departments such as Medicine, Pharmacy, Anaesthesia, Nursing, Medical laboratory, Public Health, and Midwifery.

A university-based cross-sectional survey study was conducted to evaluate students’ knowledge, attitudes, and practices about the usage and resistance at Debre Tabor University in Northwest Ethiopia. The researcher's exploration of relationships between variables like knowledge, attitudes, and practices related to antibiotic usage is made possible by cross-sectional studies, which are especially well-suited for KAP assessments because they offer data from a large population at a single moment (Ishtiaq, 2019).

### Study population and eligibility criteria

Regular undergraduate students who were enrolled at Debre Tabor University during the study period made up the study population. Participation was deemed possible for all available students who gave their freely informed consent. Excluded were students who were very sick or who had cognitive, language, or communication difficulties that made it difficult for them to comprehend the study's goals or give their informed permission. This strategy upheld the ethical norms for voluntary participation while guaranteeing that people could actively participate in the study.

### Sample size determination and sampling technique

The study participants included in the study was determining by using the single population proportion formula, n  = $\frac{\;{\left(\mathrm{Z\alpha}/2\right)}^{2}P(1-P)}{d^{2}}$

Where: **n** = Minimal sample size required.

**Z 2 a/2** = Standard normal deviation at 95% confidence interval corresponding to 1.96.

***P*** **=** the prevalence of knowledge of antibiotic use and

**d** = the margin of error, a 5% margin of error.

The *P* value was taken using data from an earlier study conducted at the University of Gonder, 75.1% (Seid & Hussen, 2018a). This yielded a sample size of 287. After adding a 10% contingency for non-responses, the final sample size was 316. Participants were selected using a simple random sampling technique.

The study participants were randomly selected using a stratified random sampling method. First, students were stratified by department and by academic year to gain proportional representation from all faculties. Next, simple random sampling was used for each stratum following a list of students obtained from the university registrar. This sampling method allows for the equal probability of selection for all eligible students to notice representativeness and reproducibility followed by periodic sample sampling.

### Definition of terms and operational definition

**Attitude**: Feeling toward the subject, including judgment of its importance and influence on people’s lives (Abdul Halim et al., 2020).

**Knowledge:** The degree of factual understanding of the topic and associated issues (Harstad et al., 2022).

**Practice:** Current action taken; as a result, knowledge, attitude, and perception toward the issue (O’Neill, 2016).

**Good knowledge:** Students were deemed to possess good knowledge if they answered half or more of the knowledge questions (Baig et al., 2020).

**Favourable attitude:** Students were classified as having a positive attitude if they answered at least half of the questions about attitude correctly (Shaka & Senbeto, 2018).

**Good practice:** Students were considered to have good practices if they responded to half or more of the practice-related questions (Bolarinwa, 2015).

### Data collection tools, procedures, and measurement

A variety of literature sources were used in the preparation of the structured questionnaire (Barakh, et al., 2016; Battah, Malaysia, et al., 2021; Gupta et al., 2017; Scaioli et al., 2015; Seid & Hussen, 2018b; World Health, 2012). The data collection tool was evaluated for face validity, completeness, and content clarity and was approved by two senior clinical pharmacists who are academicians and researchers. After obtaining informed consent from each student, data were collected through in-person interviews with a questionnaire. The questionnaire was initially prepared in English, translated into Amharic during the interviews, and subsequently back-translated into English during the interpretation process and analysis. Data were collected using printed copies of a structured questionnaire through interviewer-administered, face-to-face interviews. Trained healthcare professionals with prior experience in data collection served as interviewers. All interviewers were trained on the study's goals, the questionnaire's content, and pertinent ethical issues prior to data collection in order to maintain uniformity and reduce interpretation bias. The questionnaires were divided into three sections: the first examined sociodemographic factors such as age, sex, faculty, and educational level; the second examined knowledge, attitudes, and practices related to the use of antibiotics. Nine knowledge questions evaluated students’ awareness of the use of antibiotics; six attitude questions evaluated students’ attitude toward the use of antibiotics; and eight practice questions evaluated students’ practices regarding the use of antibiotics. The third one was design knowledge and attitude regarding to antibiotic resistance. There are ten questions to gauge knowledge about antibiotic resistance and five questions to gauge attitude toward antibiotic resistance. Data were collected from students enrolled in health-related majors (HRMs) and non-health-related majors (NHRMs).

### Data quality control

The questionnaire was pretested on 5% of the study participants among Debre Tabor University extension students over the weekend to ensure clarity and comprehensibility. Based on the pretest results, minor wording adjustments were made to improve question clarity and flow. Subsequent to the initiation of the data collection process, all data were securely stored in a designated location to maintain confidentiality and data integrity. Frequent and timely supervision of data collectors was undertaken by the supervisors and principal investigator.

### Data processing and analysis

The Statistical Package for Social Science for Windows version 27 was used to enter and analyze the data. Categorical variables were described by frequencies and percentages, and data were expressed in the form of tables, and texts described based on the characteristics of the data. To identify factors associated with KAP regarding antibiotic use, both bivariable and multivariable logistic regression analyses were performed. Initially, bivariable logistic regression was used to assess the association between each independent variable and the KAP outcomes. Variables with a *p*-value < 0.25 in the bivariable analysis were included in the multivariable logistic regression model to control for potential confounders. Model fitness was assessed using the Hosmer-Lemeshow goodness-of-fit test. A *p*-value < 0.05 was considered statistically significant in the final multivariable model.

### Ethics approval and consent to participate

The College of Health Science at Debre Tabor University’s research and ethical review committee, with approval number 1614/2022, was obtained before initiating any data collection. Written informed consent was obtained from each participant, and the study's objectives were made evident to the students before the start of data collection. The participants’ rights to decline or end the interview at any time were maintained, and no questions about disclosing student identity or name were posed. The study was conducted in accordance with the principles of the Declaration of Helsinki, ensuring strict adherence to anonymity and confidentiality throughout the research process.

## Results

### Socio-demographic characteristics

A total of 316 participants were included in the study, with males comprising the majority at 176 (55.7%). The majority (71.5%) of the students were between 20 and 25 years old. About 177 (56.0%) of the students were coming from rural. 215 (68.0%) were from non-health-related major faculty and the remaining were from health-related colleges for both groups, the largest proportion of students were second-years (53 in health-related majors (HRMs)) and 99 in non-health-related majors (NHRMs) respectively (Table 1).

**Table 1.** Sociodemographic characteristics of antibiotics use and resistance among regular undergraduate students at DTU, Ethiopia, from April 1 to June 30, 2022 (n = 316).

Variable	Category	Frequency	Percentage (%)
Sex	Male	176	55.7
	Female	140	44.3
Age in year	<20	14	4.4
	20–25	226	71.5
	> = 26	76	24.1
Resident	Rural	177	56.0
	Urban	139	44
Educational level	1st Year	55	17.4
	2nd Year	97	30.7
	3rd Year	102	17.1
	4th Year	55	17.4
	5th Year	7	17.4
Faculty	HRMs	101	32.0
	NHRMs	215	68.0
Antibiotics use within the last 12 months	Yes	170	53.8
	No	146	46.2

HRMS – health-related majors, NHRMs – non-health-related majors.

### Knowledge of students about antibiotic use and resistance

#### Knowledge of students’ on antibiotic use

Concerning the responses to the eight questions about the use of antibiotics; at least 74% of the participants correctly answered the included questions. Antibiotics are effective against bacteria 77.5%, and they can cause adverse drug reactions 74%, A majority of participants (77.5%) disagreed with leftover antibiotics can be kept at home for future needs for oneself or others, and Antibiotics should not be purchased and taken without a doctor’s prescription 78.5%. On the other hand, four out of the eight questions were correctly answered by fewer than 33% of the participants. Over half (52.4%) of the students had better knowledge regarding antibiotic use (Table 2).

**Table 2.** Knowledge of antibiotic use among regular undergraduate students at Debre Tabor University, Ethiopia, from April 1 to June 30, 2022 (n = 316).

No	Statement	Agree	Disagree	Not sure	Correct answer
		N (%)	N (%)	N (%)	N (%)
1	Common colds are cured more with antibiotics	205 (64.9)	60 (19)	51 (16.1)	60 (19)
2	Antibiotics are effective against bacteria	245 (77.5)	26 (8.2)	45 (14.3)	245 (77.5)
3	Antibiotics are effective against viruses	42 (13.3)	83 (26.3)	191 (60.4)	83 (26.3)
4	Ear infections in children 3 – to 6 years old almost always require antibiotics	129 (40.8)	181 (57.3)	6 (1.9)	18 1(57.3)
5	Antibiotics can cause adverse drug reactions	234 (74.0)	63 (19.9)	19 (6.1)	234 (74.0)
6	Leftover antibiotics can be kept at home for future needs for oneself or others	47 (14.9)	245 (77.5)	24 (7.6)	245 (77.5)
7	Antibiotics should not be purchased and taken without a doctor’s prescription	248 (78.5)	16 (5)	52 (16.5)	248 (78.5)
8	Antibiotics can be stopped before the prescription is finished if the patient feels better	156 (49.4)	105 (33.2)	55 (17.4)	105 (33.2)
9	Antibiotics can reduce pain or fever	216 (68.4)	89 (28.2)	11 (3.4)	89 (28.2)

Statements 2, 5, 7 are correct = yes (agree) and Statements 1, 3, 4, 6, 8, 9 are correct = no (disagree and not sure); the appropriate response for each statement is shown in the ‘Correct Answer’ column.

#### Knowledge of students on antibiotic resistance

Regarding students’ knowledge of antibiotic resistance, over two-thirds (63.6%) recognised that one of the biggest issues facing the globe now is antibiotic resistance. Over half (54.7%) of those with sufficient understanding of antibiotic resistance, while 24.8% stated they knew nothing at all, and 20.5% said they had no idea (Table 3).

**Table 3.** Knowledge level of regular undergraduate students regarding antibiotic resistance at DTU, Ethiopia from April 1 to June 30, 2022 (n = 316).

No	Statement	Yes	No	Do not know	Correct answer
		N (%)	N (%)	N (%)	N (%)
1	Bacteria can become resistant to antibiotics	213 (67.4)	60 (19.0)	43 (13.6)	213 (67.4)
2	People can become resistant to antibiotics.	97 (30.7)	152 (48.1)	67 (21.2)	152 (48.1)
3	The more antibiotics are used in society, the higher the risk that resistance develops and spreads.	126 (39.9)	85 (26.9)	105 (33.2)	126 (39.9)
4	Non-compliance does not contribute to the development of antibiotic resistance.	79 (25.0)	145 (45.9)	92 (29.1)	145 (45.9)
5	Resistance can spread from animals to humans.	131 (41.5)	87 (27.5)	98 (31.0)	131 (41.5)
6	Resistance can spread from person to person.	203 (64.2)	46 (14.6)	67 (21.2)	203 (64.2)
7	Today, antibiotic resistance is not a big problem in the world.	24 (7.6)	201 (63.6)	91 (28.8)	201 (63.6)
8	Taking antibiotics correctly may reduce the risk of antibiotic resistance	216 (68.4)	38 (12.0)	62 (19.6)	216 (68.4)
9	Antibiotics should be stopped immediately when the patient is clinically improved to reduce the risk of resistance	67 (21.2)	151 (47.8)	98 (31.0)	151 (47.8)
10	Students have a role in decreasing resistance?	187 (59.2)	67 (21.2)	62 (19.6)	187 (59.2)

Knowledge statements 2, 4, 7, and 9 are false; the appropriate response for each statement is shown in the ‘Correct Answer’ column.

### The attitude of students on antibiotic use and resistance

#### Attitudes toward antibiotic use

Regarding attitude toward antibiotic use, half (50.0%) of participant students agreed with the statement ‘Antibiotics are safe drugs; hence, they can be used commonly.’ ‘The highest number of students (64.2%) believed that receiving information about antibiotics is necessary.’ A majority of students, 91 (28.8%), disagreed with the statement that ‘always taking more than one antibiotic at a time is good for treating diseases.’ A small percentage of students, 11 (4.1%), were undecided about the statement ‘Doctors conduct thorough examinations to determine whether a patient needs antibiotics or not. Approximately 48.9% of students expressed a favourable attitude regarding the use of antibiotics’ (Table 4).

**Table 4.** Attitudes toward antibiotic use among regular undergraduate students at Debre Tabor University, Ethiopia from April 1 to June 30, 2022 (n = 316).

No	Statement	Agree	Neutral	Disagree
		N (%)	N (%)	N (%)
1.	Antibiotics are safe drugs; hence, they can be used commonly	158 (50.0)	68 (21.5)	90 (28.5)
2.	It is necessary to get more information about antibiotics.	203 (64.2)	57 (18.0)	56 (17.8)
3.	Doctors run thorough examinations of whether a patient needs antibiotics, or not.	191 (60.4)	57 (18.0)	68 (21.6%)
4.	Always taking more than one antibiotic at a time is good for treating diseases.	164 (51.9)	61 (19.3)	91 (28.8)
5.	Antibiotics are effective for all types of infections.	172 (54.4)	65 (20.6)	79 (25.0)
6.	Physicians often prescribe antibiotics unnecessarily.	143 (45.2)	66 (20.9)	107 (33.9)

‘Agree’ favourable attitude; ‘neutral and disagree’ a non-positive or unfavourable attitude.

#### Attitude towards antibiotic resistance

Regarding students’ attitudes toward ABR, less than one-third (28.5%) believed that improper antibiotic use is a significant contributing factor to the emergence of antibiotic resistance. The largest proportion of respondents, 171 (54.1%), were neutral regarding the statement that ‘skipping one or two doses of antibiotics does contribute to the development of antibiotics resistance. Furthermore, 235 students (74.4%) incorrectly believed that antibiotic resistance is increasing due to the effectiveness of antibiotics, reflecting a misunderstanding of the concept. Nearly half (45.2%) of the students expressed favourable attitudes toward antibiotic resistance (Table 5).

**Table 5.** Attitude towards antibiotic resistance among regular undergraduate students at DTU, Ethiopia from April 1 to June 30, 2022 (n = 316).

No	Statement	Agree	Neutral	Disagree
		N (%)	N (%)	N (%)
1.	Inappropriate use of antibiotics is a major factor that bacteria can resist antibiotics.	104 (32.9)	184 (58.2)	42 (13.3)
2	Skipping one or two doses of antibiotics does contribute to the development of antibiotic resistance.	96 (30.4)	171( (54.1)	41 (13.0)
3	Antibiotic resistance can affect you and your family’s health.	89 (28.2)	176 (55.7)	44 (13.9)
4.	Antibiotic resistance is a problem in our country.	81 (25.6)	171 (54.1)	56 (17.7)
5.	Antibiotic resistance is increasing	81(25.6)	195 (61.7)	40 (12.7)

### Students practice on antibiotic use

Eight practice questions regarding the use of antibiotics were addressed by the participants’ answers. A total of 43.7% of participants reported that they always completed the full course of antibiotics prescribed by their doctor, 16.5% stated that they never missed a dose, and 21.8% reported that they never shared their medication with family members or others who had similar symptoms (Table 6).

**Table 6.** Practice of antibiotic use among regular undergraduate students at Debre Tabor University, Ethiopia from April 1 to June 30, 2022 (n = 316).

No	Statement	Always	Sometimes	Rarely	Never
		N (%)	N (%)	N (%)	N (%)
1.	I am taking the antibiotic directly from the pharmacists without the need for a prescription from a doctor	64 (20.3)	98 (31.0)	57 (18.0)	97 (30.7)
2.	I usually keep leftover antibiotics at home for future need	43 (13.6)	85 (26.9)	79 (25)	109 (34.5)
3.	I usually complete the antibiotic course for the period described by my doctor	138 (43.7)	75 (23.7)	36 (11.4)	67 (21.2)
4.	I usually do not miss any of the doses while completing the course of antibiotic	167 (52.8)	35 (11.1)	62 (19.6)	52 (16.5)
5.	If I feel better after a few days, I usually stop taking my antibiotics before completing the course of treatment	64 (20.3)	141 (44.6)	42 (13.3)	69 (21.8)
6.	I do not share antibiotics with someone else in my/our family/friends with similar symptoms	81 (25.7)	75 (23.7)	91 (28.8)	69 (21.8)
7.	I usually ask the doctor for an antibiotic prescription once I or one of my family members have a sore throat or fever and cough	48 (15.1)	95 (30.1)	103 (32.6)	70 (22.2)
8.	I do not usually see another doctor if he/she does not prescribe antibiotics	46 (14.6)	77 (24.4)	94 (29.7)	99 (31.3)

### Factors associated with knowledge, attitudes, and practices relating to antibiotic use

Multivariate logistic regression was performed for variables with *p*-values less than 0.25 following the completion of univariate analysis. Following the adjustment for other variables, the multivariable logistic regression analysis revealed that females compared to men have a 1.7-fold increase in the probability of using antibiotics practices (AOR =   1.720, 95% CI = 1.271 - 3.829, *p* = 0.023). Rural students were 48% less likely than those in urban areas to be aware of the use of antibiotics by 48% (AOR =   0.526, 95% CI = 0.159 - 0.837, *P* = 0.012). Compared to students in health-related majors, those in non-health-related majors demonstrated poorer knowledge (34%) and poor practice (45%) regarding antibiotic use, with adjusted odds ratios of 0.660 (95% CI: 0.159–0.987, *P* = 0.012) for knowledge and 0.551 (95% CI: 0.292–0.845, *P* = 0.013) for practice, respectively (Table 7).

**Table 7.** Logistic regression analysis of factors associated with poor knowledge, unfavourable attitudes, and unfavourable practices of AMU among Debre Tabor University students, in Ethiopia.

Variables	Categories	Knowledge level		Attitudes level		Practices level	
		AOR (95% CI)	*P*- value	AOR (95% CI)	*P*- value	AOR (95% CI)	*P*- value
Sex	Male	1		1		1	
	Female	0.793(0.391–1.759)	0.614	1.571 (0.624–2.931)	0.34	1.720 (1.271–3.829)	0.023*
Age	<20	0.351 (0.101–0.627)	0.011*	0.246 (0.098–0.471)	0.010*	0.120 (0.083–0.293)	<0.001*
	20–25	0.385 (0.120–0.806)	0.043*	0.457 (0.210–0.712)	0.013*	0.255 (0.081–0.937)	<0.001*
	> = 26	1		1		1	
Residence	Rural	0.526 (0.159–0.837)	0.012*	0.459 (0.165–1.602)	0.524	0.455 (0.292–1.145)	0.135
	Urban	1		1		1	
Education	≤2^nd^ Year	0.271 (0.060, 1.212)	0.088	0.376 (0.95, 1.492)	0.164	1.272 (0.313, 5.163)	0.736
	3^rd^ Year	2.714 (0.861, 8.558)	0.088	1.377 (0.480, 3.945)	0.552	1.027 (0.324, 3.256)	0.964
	4th Year	1.837 (0.741, 4.558)	0.189	0.669 (0.278, 1.611)	0.370	1.089 (0.430, 2.756)	0.858
	≥5th Year	1		1		1	
Faculty	HRM	1		1		1	
	NHRM	0.660 (0.159–0.987)	0.012*	1.459 (0.165–1.602)	0.244	0.551 (0.292–0.845)	0.013*
Antibiotic use in the last 6 months	Yes	0.526 (0.159–0.837)	0.025*	0.459 (0.165–1.602)	0.524	0.455 (0.292–1.145)	0.135
	No	1		1		1	

AOR: Adjusted Odd Ratio CI: Confidence Interval, *p*-value < 0.05: Statistical significant, HRMS – health-related majors, NHRMs – non-health-related majors.

## Discussion

Antimicrobial resistance, especially antibiotic resistance, is one of the major global public health threats, and is greatly facilitated through inappropriate use of antibiotics. Improving knowledge, attitudes, and practices and implementing appropriate antimicrobial use can help to mitigate this threat (Ebrahim et al., 2016; Scaioli et al., 2015). Although there is a growing research base in Ethiopia, comprehensive information on university students’ KAP for antibiotic use and resistance is limited. This study evaluated KAP for antibiotic use and resistance among convocation regular university students, while identifying key patterns and determinants.

In our survey, 78.5% of participants felt that antibiotics should not be acquired or used, without a prescription from a physician, which is consistent with 75% reported in (Alkhalifah et al., 2022), and higher than 62% (El Zowalaty et al., 2016). Differences in the way studies reported results may be related to differences in education levels, professions, and access to health information (Scaioli et al., 2015). In this study, 53.5% of participants had good knowledge and 28.2% had excellent knowledge, which is greater than the previous study which assessed knowledge and noted only 43.9% of participants had good knowledge (Alkhalifah et al., 2022). We also reported apparently similar findings in Nigeria (Ayepola et al., 2019), the University of Gonder (Dejene et al., 2022), and Bangladesh (Azim et al., 2023) supported this conclusion; however, a study from Sudan (Sunusi et al., 2019) did not support it. The difference may be due to varying levels of focus on infectious diseases and antibiotic use in the students’ curricula or personal interest, which can affect their knowledge.

Regarding awareness of antibiotic resistance, 54.7% of participants were sufficiently aware, in line with Jordan (50%) (Darwish et al., 2014), but higher than 31.9% (Fetensa & Wakuma, 2020) and the 39.6% (Chaw et al., 2018), and lower than Eastern Ethiopia (78%), and Namibia. (72%) (Jifar & Ayele, 2018; Pereko et al., 2015). This difference may be due to differences in regulations and their implementation or other contextual factors beyond mere knowledge and beliefs.

The finding was that about half of students (50%) thought antibiotics were safe, which is somewhat lower than the 59.4% reported in Colombia (Higuita-Gutiérrez et al., 2020). As a comparison 60% of students had a positive perspective of antibiotic use, while 48.9% had negatively inclined attitudes in Sudan (Sunusi et al., 2019). The neutral responses in this study illustrate knowledge deficits and lack of coherent instruction, and support the need for educational interventions related to antibiotic use and recognition of resistance.

Antibiotic resistance has been extensively discussed and publicised in the press, media, and online discussion forums, and the public is becoming knowledgeable about the burden of resistance. The study found that a large proportion of individuals had negative attitudes toward antibiotic resistance, with only 45.2% having positive attitudes towards antibiotic resistance, which is lower than a study conducted at the Indian and University of Gonder that found 73.5 and 96% respectively (Dejene et al., 2022; Khan et al., 2013). The result of variations brought forth by media attention, healthcare systems, public awareness initiatives, and educational level. These disparities in opinions among locations are probably also influenced by cultural and socioeconomic variables, as well as individual experiences with antibiotic resistance.

This research showed that 51.3% of students completed the entire prescribed course of antibiotics, while 43.7% followed the physician-specific use. This is consistent with the finding showing that only half of the participants had belief that a prescription was needed. In other situations, participants had higher use of antibiotics without a prescription: India (76%) (Nguyen et al., 2022); 91.7% in Bangladesh (Azim et al., 2023), 56.0% in Nigeria (Ayepola et al., 2019), and 69.2% in Debre Markos (Zelellw & Bizuayehu, 2016). These differences may be dependent on education, socio-demographics, and healthcare access. Another significant finding was that 13.6% of students reported storing leftover antibiotics at their home. This may increase improper use of antibiotics and antibiotic resistance (Cantón et al., 2022), especially in rural areas with limited healthcare. This supports the need to consult healthcare providers before using antibiotics, and to pursue educational initiatives to show the risks of using prescription drugs without a prescription.

The results indicate that socio-demographic factors such as age, residence, faculty, and recent awareness of antibiotic use significantly influenced students’ knowledge. Younger students may have had less exposure to educational materials, impacting their understanding of antibiotics (Shrestha, 2019). Additionally, since they have easier access to healthcare information than students from rural areas, students from urban areas can be more aware (Ajzen & Fishbein, 2000; Kumar et al., 2020). Faculty distinctions are also important; students in health-related fields may receive more in-depth instruction on antibiotic stewardship than students in other subjects (Liu et al., 2021). Recent awareness regarding antibiotic use is also crucial because it indicates continued education and interest in health-related issues, which can improve memory retention and responsible use practices (Sakeena et al., 2018). On the other hand, age was the sole socio-demographic variable linked to attitudes toward antibiotic use. Nonetheless, prior studies have demonstrated that older respondents, females, who had finished college, had more education, or were employed in the healthcare sector were more likely to have better attitudes, knowledge, and better practices about the use of antibiotics(Karuniawati et al., 2021; Nepal et al., 2019).

Our research revealed that the socio-demographic factors that were associated with the practice of using antibiotics were sex, age, and faculty. The results match the ones that were published (Gebeyehu et al., 2015; Nepal et al., 2019). Frequent use of antibiotics are a major contributor to ABR, particularly when they are utilised improperly (Bell et al., 2014; Chung et al., 2007). Since a recent thorough study indicated that, there is a significant likelihood of multidrug use and antibiotic resistance (Muhie, 2019). Males, younger respondents, and married respondents were linked to inappropriate use of antibiotics (Yu et al., 2014).

Results were indicative of non-health majors being lower in knowledge and practices than health-related students, which is likely representative of the broader receive training on health care of health students, and therefore are more knowledgeable of antibiotic resistance (Zhong et al., 2020). Through this formal teaching, a better understanding of appropriate antibiotic use and its importance in clinical practice can be obtained. Students who study subjects unrelated to health may not be exposed to these important subjects, whereas; students studying health-related subjects are often engaged in discussions and case studies that emphasise the importance of antibiotic stewardship (Fitzgerald & Palincsar, 2019).

Similar to Debre Tabor University, a number of strategies are suggested to improve university students’ understanding, attitudes, and practices about antibiotic use and resistance. First, putting in place focused educational initiatives can greatly increase awareness; seminars and workshops that address the causes of antibiotic resistance, proper use, and the dangers of abuse can efficiently educate students (Zeb et al., 2022). Peer-led programs can also have a significant effect because students can relate to one another more easily and find material that is more interesting (Fitzgerald & Palincsar, 2019). Including courses on antibiotic stewardship in university curricula can also guarantee that all students have a basic understanding of this important topic, particularly in faculties that deal with health (Marvasi et al., 2021). In general, these initiatives can support college students in creating an awareness and responsibility culture about antibiotic use.

## Limitations of the study

This research has a number of limitations that should be acknowledged when interpreting the results of this study. First, due to the cross-sectional design of the study, KAP is only captured at one time point which does not allow for causal inferences or an examination of any temporal trends. Second, while questionnaire data is valuable, self-report data also has potential risks for social desirability bias as participants may over report desirable behaviours or knowledge. Third, while the sample size is robust, data were only collected from a single university, which reduces generalizability to other students from other institutions, regions, or countries that may have different curricula, access to healthcare, or cultural norms. Finally, possible response bias and the social desirability aspect is also a potential confounding variable which should be noted due to a potential influence of the interviewer on participants’ answers if participants were interviewed while administering the questionnaires.

## Conclusion

The study revealed that inadequate knowledge, unfavourable attitudes, and improper practices among students regarding antibiotic use and antibiotic resistance. Factors such as age, gender, and rural origin, major unrelated to health, and uses of antibiotics over the previous six months were associated with the KAP of antibiotic use. These findings provide insight into students’ understanding of antibiotic resistance, which will aid initiatives to increase public awareness of the issue and its solutions. Training programs, instructional materials, and meetings could all be used to implement intervention designs. By concentrating on these demographics, the project hopes to yield informative data that can impact public health and educational programs in Ethiopia and other similar settings. Policymakers and healthcare professionals can use the information from this study to create focused education campaigns that will lessen the abuse of antibiotics and fight resistance.

## Data Availability

Upon reasonable request, any data related to this study can be obtained from the corresponding author.
